# Influence of social support on subjective well-being of patients with chronic diseases in China: chain-mediating effect of self-efficacy and perceived stress

**DOI:** 10.3389/fpubh.2023.1184711

**Published:** 2023-06-21

**Authors:** Zhenni Luo, Sisi Zhong, Siyu Zheng, Yun Li, Yan Guan, Weihong Xu, Lu Li, Siyuan Liu, Haozheng Zhou, Xuanhao Yin, Yibo Wu, Diyue Liu, Jiangyun Chen

**Affiliations:** ^1^School of Health Management, Guangzhou Medical University, Guangzhou, China; ^2^School of Public Health, Southern Medical University, Guangzhou, China; ^3^School of Public Health, Peking University, Beijing, China; ^4^International School of Public Health and One Health, Hainan Medical University, Haikou, China; ^5^Center for WHO Studies and Department of Health Management, School of Health Management of Southern Medical University, Guangzhou, China

**Keywords:** social support, self-efficacy, subjective well-being, perceived stress, chain-mediating effect, patients with chronic diseases

## Abstract

**Introduction:**

The well-being of patients with chronic diseases is an issue of widespread concern in public health. While social support is thought to have a positive effect on it, the mechanisms of its influence have not been fully addressed. Thus, we explored the possible mediating effects of self-efficacy and perceived stress to determine the relationship between social support and well-being in these patients.

**Methods:**

A cross-sectional study was conducted among 4,657 patients with chronic diseases in China. The PROCESS Macro model 6 of SPSS was employed to explore the intermediary role between variables.

**Results:**

Self-efficacy and perceived stress played a partial intermediary role between social support and subjective well-being, with an effect ratio of 48.25% and 23.61%, respectively. Self-efficacy and perceived stress had a chain intermediary effect (28.14%) between social support and subjective well-being.

**Discussion:**

This study suggested that improving the self-efficacy of patients with chronic diseases to cope with the changes in social support caused by the disease could reduce stress and enhance subjective well-being.

## 1. Introduction

Non-communicable diseases (NCDs), also known as chronic diseases, tend to persist over long durations. The main types of NCDs are cardiovascular diseases, cancers, chronic respiratory diseases, and diabetes, which are the biggest killers globally (1). Due to the industrial environment and modern lifestyle, the number of people with chronic diseases is growing rapidly in the 21st century, and the onset age is becoming younger. According to a World Health Organization’s (WHO) report released in 2021, the global chronic disease death rate increased from 60.8% in 2000 to 73.6% in 2019 (2). From 2010 to 2040, it is predicted that the proportion of people aged 60 years plus in China will increase from 12.4% (168 million) to 28% (402 million) (3). According to the China Research Centre on Aging, an estimated 202 million people aged 60 years and older were living in China in 2013, of whom up to 50% were suffering from chronic NCDs (4). Because of the associated heavy burden, chronic diseases have attracted global attention. In 2019, world leaders adopted a political declaration that contained a strong commitment to help relieve the burden of chronic diseases, requiring the WHO to take action to support national efforts. In May 2022, the WHO announced the goal of global chronic disease prevention and control at the 75th World Health Assembly and accelerated the process of global non-communicable disease prevention and control.

The most notable feature of chronic diseases is their long course. With the advances in medicine, the survival rate of patients with chronic diseases has increased and the mortality rate has decreased, which means that the course of the disease is further prolonged and patients live longer with illness. According to a WHO report (2) released in 2021, the global premature mortality rate due to chronic diseases dropped from 22.9% in 2000 to 17.8% in 2019. In addition, from 1990 to 2019, the disability-adjusted life years caused by chronic diseases in China increased by 8.1% (5).

In the state of living with illness, the outlook of the physiological-psychological-social health of patients is not optimistic. For example, patients with cancer may need to endure radiotherapy and chemotherapy, long-term pain, and recurrent anxiety. Patients with diabetes need to control their diet by abstinence or long-term insulin injection. Patients with cardiovascular diseases need to take medicine for a long time, restrict excessive physical labor, and so on. A large number of studies have confirmed that patients with chronic diseases for an extended period are prone to unpleasant psychological experiences, such as anxiety, depression, and other psychological problems (6). Beyoğlu and Avci (7) found that the anxiety level of adults with diabetes or hypertension was higher than that of patients with non-chronic diseases. Patients with cancer tend to have increased symptoms of anxiety and depression (8). All this brings inconvenience to the work and life of patients with chronic diseases. Therefore, in the treatment of chronic diseases, we must monitor and treat the subjective experience of long-term illness in addition to addressing recovery and death.

Subjective well-being is the overall evaluation of an individual’s quality of life according to their standard (9). It benefits individuals in many aspects, such as health and longevity (10), work and income, and relationships (11). The happiness of patients affected with chronic diseases is generally lower than that of those without diseases. Studies of older adults in Australia have shown that factors such as better subjective well-being are related to a lower possibility of chronic diseases (12). The uncertainty of the disease in patients with cancer is significantly related to their happiness level (13). To improve the state of patients with chronic diseases it is necessary to understand the factors that affect their well-being. A survey of the literature has revealed that interpersonal support, complications, and other factors have been proven to affect the well-being of patients with chronic diseases (14). Positive social support can help maintain well-being in chronic illness (15). However, the current research on the impact of social support on the well-being of patients with chronic diseases has several deficiencies. First, there is a lack of path analysis. The question of how social support affects the well-being of patients with chronic diseases is controversial. Second, the existing data sources of path analysis and research are generally limited to small regional surveys, lacking national samples.

Therefore, to address this research gap, the current study used the life data of the entire population of China to investigate the impact of social support on the well-being of patients with chronic diseases and to explore the intermediary factors that affect the process. We aimed to clarify the path of social support affecting the well-being of patients with chronic diseases to provide ideas for improving these patients’ welfare.

Social support refers to the material and spiritual support and help provided by the members of the social network closely related to the individual, and it is an essential factor affecting the sense of well-being (16). Evidence from both empirical and meta-analyses studies suggests a positive correlation between the social network of the older adults and subjective well-being (17, 18). Support from relatives, peers, and others can improve the subjective well-being of patients with chronic diseases. Research shows that peer support can improve individual health as well as the subjective well-being of patients with chronic diseases (19). People with higher social support also reported higher subjective well-being, greater life satisfaction, more positive life effects, and fewer negative life effects (20). Additionally, getting more support from family relationships can be instrumental in significantly improving the subjective well-being of the older adults (21). Children’s financial and emotional support can positively affect the subjective well-being of older adults (22). A randomized controlled experiment showed that peer mentor interventions might have merit in enabling survivors with advanced cancer to cope with their disease (23). In summary, it has been demonstrated that different types of social support provide material and emotional help for patients with chronic diseases, thus alleviating the unpleasant feelings that chronic conditions may bring while increasing the sense of pleasure.

Self-efficacy refers to people’s confidence or belief in their ability to achieve their behavioral goals in a specific field; social persuasion, physiological, and emotional state were proven to be the main factors that affect individual self-efficacy (24). Support and companionship from friends and family can contribute to a feeling of happiness, facilitate patient reconciliation with chronic illnesses, and play a role in social interaction. Research shows that among older prisoners with chronic diseases, higher emotional support, especially from clinicians, predicted higher self-efficacy (25). The role of social support showed an indirect positive effect of 58.8% on patient activation through self-efficacy (26). Peer-Led Pain Management Programs may diminish chronic pain and enhance patient self-efficacy (27). Strong self-efficacy is a factor that enables people’s happiness and success in many different occupations (28). A study on patients with spinal cord injury showed that self-efficacy, social support, and resilience were found to be significantly associated with subjective well-being (29). To further examine self-efficacy’s mediating role in social support and subjective well-being, Ie and Lee (30) found that older adults in a welfare center of Taiwan Province, China could use institutional interpersonal support to improve self-efficacy and significantly improve subjective well-being. A recent study of online learning during the COVID-19 pandemic also showed that learning self-efficacy plays an intermediary role between college students’ social support and subjective well-being (31). Therefore, social support may act on self-efficacy and may indirectly affect an individual’s subjective well-being.

Stress is the interplay of a person with their environment that the individual deems as menacing or affecting one’s potential, resources, or well-being (32). As a negative life event, chronic disease may cause patients to experience a variety of negative pressures, such as the financial pressure associated with medical expenses and social pressure associated with disease stereotypes. Stress is considered to be closely related to the occurrence and development of some chronic diseases (33). Fatigue and debilitation symptoms of chronic diseases are also seriously affected by perceived stress (34). Studies have shown that social support is a critical factor affecting the stress symptoms of patients with chronic diseases (35). Long-term perception of high stress shapes negative cognition, brings about negative emotions, and affects normal physiological functions of the human body (36). Notably, social support such as family financial support and doctor’s treatment guidance can reduce the perceived stress of patients with chronic diseases. Loayza-Rivas and Fernández-Castro (37) showed that social support, as a protective factor, could reduce the perceived stress of Peruvian immigrants, thus improving their subjective well-being.

However, self-efficacy and perceived stress have not been explored in previous studies on the effect of social support on the well-being index of patients with chronic diseases. Secondly, most empirical studies focus on individual studies of factors such as social support of the population, while there are relatively few studies on how they interact and connect with each other. Thirdly, most of the international research on the mental health of patients with chronic diseases is carried out in western countries. However, few scholars have carefully examined how self-efficacy, subjective well-being, social support and perceived stress of patients with chronic diseases interact with each other under the cultural background of China. Exploring the relationship between social support and subjective well-being of patients with chronic diseases under the background of China’s characteristic system during the epidemic period is helpful to enrich the relevant theoretical framework from different cultures and institutional groups. It can also provide a basis for local authorities to formulate policies and interventions. Thus, this study aimed to examine the influence path of social support on the well-being index of patients with chronic diseases and to provide a theoretical basis for the improvement of the well-being index of patients with chronic diseases in China. According to the above literature review, the following hypotheses were put forward:

*Hypothesis 1 [H1].* Perceived social support can positively predict the subjective well-being of patients with chronic diseases.

*Hypothesis 2 [H2].* Self-efficacy plays an intermediary role between perceived social support and well-being.

*Hypothesis 3 [H3].* Stress perception plays an intermediary role between social support and well-being.

## 2. Materials and methods

### 2.1. Participants and procedure

This research data came from a cross-sectional survey on the psychology and behavior of residents in China during the epidemic period from June 20, 2022 to August 31, 2022. The survey adopted stratified sampling and quota sampling, and selected 148 cities, 202 districts and counties, 390 townships/streets, and 780 communities/villages (excluding Hong Kong, Macao, and Taiwan) in 23 provinces, five autonomous regions, and four municipalities in China (38). In total, 30,505 people were surveyed using an electronic questionnaire. The participants in this study were patients with chronic diseases in China. Thus, 6,812 patients with chronic diseases in this survey were selected for further screening. Those individuals who answered Question 25, “Have you been diagnosed by a doctor and are you currently diagnosed with the following chronic diseases?” and the participants who chose any option other than “None” were included in the scope of this study. The chronic diseases mentioned in Question 25 include fractures, Catagma, Cataracts, Osteoporosis, Arthritis, and other conditions. According to the established questionnaire screening criteria, two researchers were selected to conduct back-to-back logic examinations and questionnaire screenings. The rejection criteria for unqualified questionnaires were (a) questionnaires answered in less than 240 s; (b) questionnaires with inconsistent logic checks; (c) incomplete questionnaires; (d) duplicate questionnaires; (e) questionnaires with the same options in the scale for setting reverse questions. After the screening, 4,657 valid questionnaires (mean age = 50.58 ± 18.91 years), including 2,493 women (53.53%), remained.

### 2.2. Research tools

#### 2.2.1. Perceived Social Support Scale (PSSS)

The Perceived Social Support Scale (PSSS) is a social support scale that emphasizes individuals’ self-understanding and self-feeling (39). It includes three seven-point Likert items (strongly disagree—strongly agree), and mainly measures various social support sources that individuals perceive from family, friends, and others (three dimensions: family support, friend support, and other support) (40). In this study, Cronbach’s alpha coefficient of this scale was 0.828.

#### 2.2.2. New General Self-Efficacy Scale (NGSES)

Self-efficacy refers to people’s self-confidence in the ability to perform and adhere to a specific behavior (41). It measures the belief of patients in their ability to achieve the desired results through persistence and effort, which can reflect their self-management level. The higher the level of self-efficacy, the higher the level of self-management. The scale consists of three five-point Likert items from 1 (completely incorrect) to 5 (completely correct). The higher the total score, the higher the self-efficacy level of the individual. In this study, Cronbach’s alpha coefficient of this scale was 0.904.

#### 2.2.3. Well-being index scale (WHO-5)

In this study, the World Health Organization’s Five Physical and Mental Health Indicators (WHO-5) Well-being Index Scale was used to investigate the subjective well-being of patients with chronic diseases. The WHO-5 well-being index is a widely used, short rating scale that measures subjective well-being (42). The reliability and validity of WHO-5 are optimal for screening the psychological well-being of patients with chronic illness (43). The participants were given five items divided into six grades according to their psychological situation during the past 2 weeks (0, “Never”; 1, “Sometimes”; 2, “Less than half of the time”; 3, “More than half the time”; 4, “Most of the time”; 5, “Always”). The scores were added together to obtain the original score. We multiplied the original score by four to obtain the final score (0 ~ 100 points), which is the subjective well-being index. The higher the total score, the healthier the mood. A score of <29 indicates “low subjective well-being,” which requires further research on psychological depression. A score of 29 ~ <50 indicates “weakened subjective well-being.” A score of ≥50 indicates “feeling happy.” In this study, Cronbach’s alpha coefficient of the scale was 0.929, which suggests that the scale has good internal consistency.

#### 2.2.4. Perceived stress scale (PSS-4)

PSS-4 is a psychological instrument for measuring a person’s perception of stress in the previous month and the degree to which life’s situations are perceived as stressful (44). Numerous studies have used the PSS to examine the relationship between stress and quality of life, depression, and other parameters (45). There are four five-point Likert items (0, never; to 4, always); the higher the score, the greater the pressure perceived by the participants. This scale has primarily been used to evaluate the perceived stress level of the participants in the past month. In this study, Cronbach’s alpha coefficient using this scale was 0.710.

### 2.3. Statistical analysis

In the current study, SPSS 25 (46) (IBM, Armonk, NY, United States) and Model 6 of PROCESS Macro (47) were used to analyze the survey data. Descriptive statistics were used to analyze the sample characteristics. Pearson correlation coefficient is used to determine the correlation among self-efficacy, perceived stress, social support and subjective well-being. Bootstrap in this study is based on 5,000 samples. For the significance test, a 95% confidence interval (CI) was used, and the significance level was set to 0.05. To further improve the rigor of the research, Harman’s single-factor test was used to examine the possible common method deviation before data analysis (48). The results revealed four factors with eigenvalues greater than 1, and the variance explained by the first factor was 26.27%, which is less than 40% of the critical standard value, suggesting no serious common method deviation problem in this study.

## 3. Results

### 3.1. General characteristics

Among the valid questionnaires, 2,164 (46.47%) were completed by men and 2,493 (53.53%) by women. There were 2,202 (47.28%) and 2,455 (52.57%) participants with agricultural and non-agricultural household registration, respectively. All participants were aged between 12 and 100 years (*M* = 50.58 years; SD = 18.91 years old); 1,235 (26.52%) completed elementary school and below, 723 (15.53%) junior high school, 881 (18.92%), technical secondary or senior high school, and 1,818 (39.04%) completed junior college and universities or above.

### 3.2. Descriptive statistics of research variables

In this study, the subjective well-being index of the participants was transformed into an ordered grade variable-subjective well-being level according to the WHO-5 scale, with 1 = low well-being (score < 29), 2 = weakened well-being (score: 29 ~ <50), and 3 = feeling happy (score ≥ 50). The total well-being of patients with chronic diseases was (*M* = 54.79, SD = 21.95) (*p* < 0.001). A descriptive analysis of the subjective well-being level of the respondents shows that most (58.88%) patients with chronic diseases are happy at present, 25.04% of the participants have decreased well-being, and 16.08% are likely to be depressed. The total social support score was 14.80 ± 3.41 (*p* < 0.001, 3 ~ 21), the total self-efficacy score was 7.58 ± 2.28 (*p* < 0.001, 0 ~ 12), and the total perceived stress score was 6.39 ± 2.77 (*p* < 0.001, 0 ~ 16) (Table 1).

**Table 1:** Descriptive statistics of research variables (*n* = 4,657).

Variable	*M* (SD)	Min–max
Subjective well-being	54.79 (21.95)	0–100
Perceived stress	6.39 (2.77)	0–16
Social support	14.80 (3.41)	3–21
Family support	5.10 (1.30)	1–7
Friends support	4.90 (1.30)	1–7
Other support	4.79 (1.35)	1–7
Self-efficacy	7.58 (2.28)	0–12
Level	2.58 (0.78)	0–4
Intensity	2.50 (0.86)	0–4
Universality	2.51 (0.84)	0–4

### 3.3. Descriptive statistics and the correlation between variables

After controlling for gender, age, agricultural household registration, and education level, a partial correlation analysis was conducted on the four research variables. The results showed that subjective well-being was significantly positively correlated with social support and self-efficacy, and negatively correlated with perceived stress, which provides preliminary evidence to support the hypotheses. In addition, self-efficacy was positively correlated with social support and negatively associated with perceived stress. However, social support is negatively correlated with perceived stress. To sum up, this study confirms that the intermediary effect can be tested. The correlation matrix, mean value, and standard deviation of each variable are shown in Table 2.

**Table 2:** Descriptive statistics and correlation analysis of variables.

Variable	1	2	3	4
1 Subjective well-being	1			
2 Social support	0.563**	1		
3 Self-efficacy	0.546**	0.611**	1	
4 Perceived stress	−0.554**	−0.434**	−0.503**	1

“**” means *p* < 0.01.

### 3.4. The mediating role of self-efficacy and perceived stress between social support and well-being

**Figure 1:**
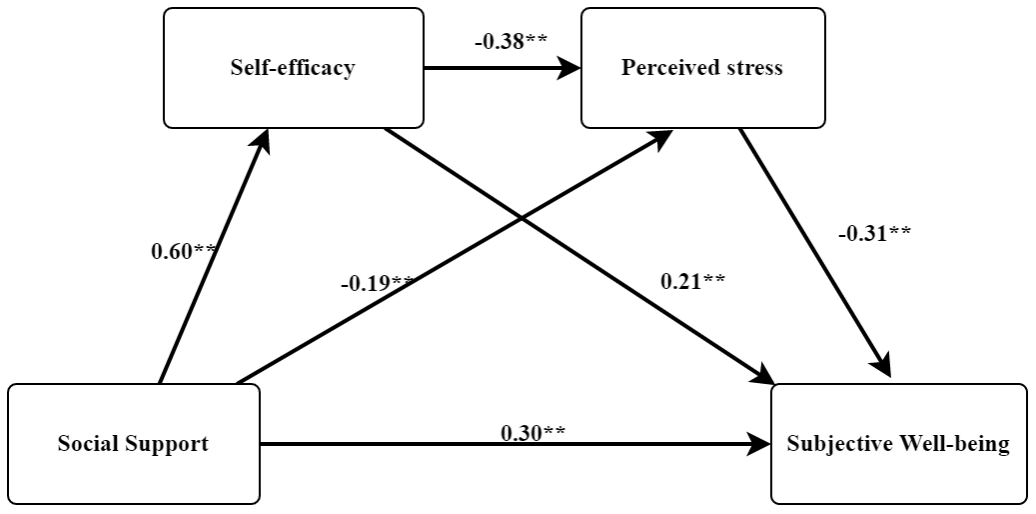
Mediating model of self-efficacy and perceived stress between social support and subjective well-being. “**” means *p* < 0.01.

In this study, the PROCESS program (Model 6) was employed for mediating effects analysis, with subjective well-being added as a dependent variable, social support added as an independent variable, and gender, household registration, age, and education level added as control variables. The total effect path coefficient of social support on well-being was significant (*γ* = 3.58, *t =* 45.92, SE = 0.08, *p* < 0.001). Second, the path model shown in Figure 1 was obtained after adding intermediary variables to the model. Taking social support (X) as the independent variable, self-efficacy (M1) and perceived stress (M2) as the intermediary variables, and subjective well-being (Y) as the dependent variable, the regression analysis was carried out.

When analyzing the mediating effects of self-efficacy and perceived stress on the relationship between social support and well-being, it was found that each model had statistical significance (Tables 3, 4, Figure 1). In step 1, social support had a significant impact on subjective well-being (*β* = 0.56, *p* < 0.001), and in step 2, social support had a significant impact on self-efficacy (*β* = 0.60, *p* < 0.001). In step 3, social support (*β* = −0.19, *p* < 0.001) and self-efficacy (*β* = −0.38, *p* < 0.001) had significant effects on perceived stress. In addition, in step 4, social support (*β* = 0.30, *p* < 0.001), self-efficacy (*β* = 0.21, *p* < 0.001), and perceived stress (*β* = −0.31, *p* < 0.001) had significant effects on participants’ depression.

**Table 3:** Moderating effect of self-efficacy and perceived stress on social support and well-being of patients with chronic disease.

No.	Variables	*β* (coeffect)	SE	*P*	95% CI		*R*^2^
					LLCI	ULCI	
1	*X→Y*	0.56	0.08	<0.001	3.43	3.73	0.33
2	*X→M1*	0.60	0.01	<0.001	0.39	0.42	0.38
3	*X→M2*	−0.19	0.01	<0.001	−0.18	−0.13	0.31
	*M1→M2*	−0.38	0.02	<0.001	−0.50	−0.43	
4	*X→Y*	0.30	0.09	<0.001	1.75	2.10	0.46
	*M1→Y*	0.21	0.14	<0.001	1.71	2.26	
	*M2→Y*	−0.31	0.10	<0.001	−2.69	−2.29	

**Table 4:** Regression analysis between table variables.

Regression equation		Overall fitting index			Significance of regression coefficient	
Outcome variable	Predictor variable	*R*	*R* ^2^	*F*	*β*	*t*
Self-efficacy	Gender	0.61	0.38	572.76	0.04	3.37**
	Age				0.00	0.06
	Household registration				−0.03	−2.45*
	Degree of education				0.06	4.02***
	Social support				0.60	51.89***
Perceived stress	Self-efficacy	0.55	0.31	343.24	−0.38	−24.62***
	Social support				−0.19	−12.51***
	Gender				−0.04	−3.21**
	Age				−0.16	−10.46***
	Household registration				0.06	4.06***
	Degree of education				0.01	0.61
Subjective Well-being	Self-efficacy	0.68	0.46	565.42	0.21	14.12***
	Perceived stress				−0.31	−24.25***
	Social support				0.30	21.62***
	Gender				−0.01	−1.28
	Age				0.05	3.95***
	Household registration				−0.00	−0.24
	Degree of education				0.01	0.72

“*” means *p* < 0.05, “**” means *p* < 0.01 and “***” means *p* < 0.001.

### 3.5. Self-efficacy, perceived stress, and the chain mediation between social support and well-being

Table 5 shows different comparisons of each path effect value to understand the significance and size of the double mediation effect. The following is the process of testing the chain mediation of self-efficacy and perceived stress between social support and well-being. The first step is to take social support (X) as the independent variable and subjective well-being (Y) as the dependent variable for regression analysis, and the regression coefficient is significant (*β* = 3.58, *p* < 0.001). Second, taking social support (X) as the independent variable and self-efficacy (M1) as the dependent variable, the regression coefficient is significant (*β* = 0.40, *p* < 0.001). In the third step, taking social support (X) and self-efficacy (M1) as independent variables and perceived stress (M2) as dependent variables make up the regression analysis, and the regression coefficient is significant (*β* = −0.16, *p* < 0.001; *β* = −0.47, *p* < 0. 001). The fourth step, taking social support (X), self-efficacy (M1), and perceived stress (M2) as independent variables and subjective well-being (Y) as dependent variables carries out the regression analysis; the regression coefficient is significant (*β* = 1.93, *p* < 0.001; *β* = 1.98, *p* < 0.001; *β* = −2.49, *p* < 0.001), the predictive effect of social support on well-being decreased significantly, and the regression coefficient decreased from 3.58 to 1.93, indicating that chain mediation was established. The ratio of mediation effect to total effect was 28.14%.

**Table 5:** Mediating effect analysis.

	Variables	Effect	Boot SE	95% CI	
				LLCI	ULCI
	Total	1.65	0.07	1.52	1.79
Indirect 1	*X→M1→Y*	0.80	0.07	0.66	0.93
Indirect 2	*X→M1→M2→Y*	0.47	0.03	0.41	0.53
Indirect 3	*X→M2→Y*	0.39	0.04	0.32	0.47
Differences (*∆B*)	Indirect 1–Indirect 2	0.33	0.08	0.17	0.48
	Indirect 1–Indirect 3	0.41	0.08	0.24	0.57
	Indirect 2–Indirect 3	0.07	0.05	−0.02	0.18

X = social support, M1 = self-efficacy, M2 = perceived stress, and Y = subjective well-being.

As shown in Table 5, each indirect effect has significant differences. In the influence of social support on well-being, we find that the mediating effect of self-efficacy is greater than perceived stress. In addition, the dual mediating effect of perceived stress and self-efficacy is greater than that of perceived stress alone, while the mediation of self-efficacy alone is more effective than the dual mediation of self-efficacy and perceived stress. In other words, self-efficacy has the most substantial mediating effect.

## 4. Discussion

### 4.1. Patients with chronic diseases have low well-being and great differences in perceived stress

In this study, the proportion of patients with chronic diseases who feel happy is slightly higher than that in Wu et al. (49). The reason may be the difference in investigation areas: in the past, scholars mainly investigated patients with chronic diseases in small areas, but the patients with chronic diseases in this study came from many provinces and cities with different economic and social development, representing chronic disease groups throughout China. Moreover, the pain caused by long-term illness can easily lead to anxiety, depression, and other negative emotions in patients with chronic diseases (50). Yet, the results of this study suggest that Chinese patients with chronic diseases have an intermediate level of well-being, which is similar to the results of a survey from 2017 (51). The longitudinal comparison demonstrated an increasing trend of the increased well-being of people with chronic diseases in China, which may be attributed to the improvement of medical care and an increase in medical resources brought about by social and economic development and the establishment of an improved medical insurance system.

### 4.2. Social support, self-efficacy, and perceived stress significantly affect the well-being of patients with chronic diseases

The results show that social support and self-efficacy can positively predict the well-being of patients with chronic diseases, whereas perceived stress can negatively predict the well-being of patients with chronic diseases. The results suggest that hypothesis H1 is valid. The higher the social support and self-efficacy and the lower the perceived pressure, the higher the happiness of patients with chronic diseases, which is consistent with the results of similar studies (10, 18, 25). Patients with chronic diseases for extended periods were more prone to debilitation (52). During this time, social support, as an individual external resource, can bring health guidance and emotional support to patients with chronic diseases, thus ameliorating patients’ bad experiences with diseases and improving their subjective well-being. Self-efficacy, as an individual’s internal resource, can automatically activate positive cognition, establish reasonable beliefs, and finally lead to health-promoting behaviors to achieve health improvement both physically and psychologically, thus enhancing the overall quality of life and subjective well-being of patients with chronic diseases. Furthermore, chronic disease stress is a type of stress feeling produced by patients in the context of the disease reality when that reality does not meet their internal health expectations. Economic pressure and even the stigma of some diseases also accompany physical weakness. Persistent chronic disease stress may induce negative emotions and organic health damage in patients with chronic diseases; thus, a lower perceived stress level could reduce adverse outcomes and improve subjective well-being.

### 4.3. The chain mediation of self-efficacy and perceived stress

The results of this study show that self-efficacy and perceived stress play a chain-mediating role between social support and well-being, accounting for 28.14%. Therefore, H2 and H3 are valid in the current study. These results indicated that improving social support could improve self-efficacy, reduce stress perception, and improve the well-being of patients with chronic diseases. The experience of self-efficacy comes from self-identification, and the initial self-identification of people comes from the outside. Therefore, as an external resource, social support can consolidate self-identification and improve self-efficacy. Patients with chronic diseases who believe that they can delay the disease’s progress or change the disease’s outcome through reasonable treatment and healthy behaviors can promote health and subjective well-being. In terms of pressure perception, this study shows that social support can reduce pressure by the following two aspects. First, instrumental support, such as social and medical security systems, pensions, family child support, and assistance with the burden of medical expenses, can reduce the economic pressure on patients with chronic diseases. Second, emotional support can reduce the emotional stress of patients with chronic diseases. For example, community health education activities and regular check-ups for chronic disease patients can reduce their worries about their physical condition. As a dimension of family support, good communication and emotional expression can reduce patients’ guilt about their diseases’ economic burden and care burden on their children. Therefore, social support can improve the well-being of patients with chronic diseases by reducing their perceived stress.

### 4.4. Strength and limitations

Compared with previous similar studies, this survey is in the period of COVID-19 epidemic, and its representative significance is different. During the epidemic period, people’s happiness and psychological problems deserve attention, and more attention should be paid to the psychological status of patients with chronic diseases. Previous studies have provided insufficient evidence to support a relationship between social support and happiness. The present study extends the existing research by providing direct evidence to prove the relationship between social support and happiness, and tests whether the possible mediating effect exists in the samples of residents with chronic diseases in China. The present study helps to clarify the relationship between social support and happiness of patients with chronic diseases in China from a more comprehensive perspective.

This study also has the following three limitations: First, this study’s data are cross-sectional; thus, it is difficult to investigate in-depth the dynamic development and changes in social support and well-being of patients with chronic diseases and the stability of their relationships. Panel data can be further used to confirm the reliability of this research conclusion in the future. Second, the samples of this study come from many provinces in China. In light of the uneven economic development in each province, the economic level of chronic disease patients may be considered a confounding factor. Future studies might consider a family’s socioeconomic status. In addition, this study did not subdivide social support as a whole, and future research can explore the effects of and compare the differences in different types of social support on the well-being of patients with chronic diseases.

## 5. Conclusion

This study demonstrated that social support could improve the subjective well-being of patients with chronic diseases. Self-efficacy and perceived stress played the mediating roles between social support and well-being, respectively, and perceived stress had a lower mediating effect than self-efficacy. Self-efficacy and perceived stress form a chain intermediary relationship between social support and subjective well-being. Self-efficacy is the proximal factor affecting the subjective well-being of patients with chronic diseases. The results of this study indicate that when promoting the well-being of patients with chronic diseases, we should consider the social support system, understand their stress, help them relieve their psychological stress, and improve the awareness of their abilities.

## Data availability statement

The datasets presented in this article are not readily available because all data generated or analyzed during this study are with the corresponding author. She is available to take any questions about the datasets. Persons who have made outstanding contributions or assisted in this study may apply for the use of the data only after submitting the study hypothesis and signing a data confidentiality agreement. There is no fee for the data opening plan. Publication of the study results will include processed data only, and personal information will remain anonymous. Requests to access the datasets should be directed to DL, L15917189871@outlook.com; JC, cjy112@i.smu.edu.cn.

## Ethics statement

The Ethics Research Committee approved the current study (No.JKWH-2022-02). The cover page of the questionnaire will explain the study’s purpose and assure anonymity, confidentiality, and the right to refuse to participate in the study. Informed consent was obtained from all participants involved in the study.

## Author contributions

ZL, SZho, and SZhe: conceptualization, validation, and writing—original draft preparation. YL, WX, YG, and LL: methodology and formal analysis. YW, DL, and JC: investigation and resources. SL, HZ, and XY: data curation. ZL and JC: writing—review and editing, supervision, and project administration. All authors contributed to the article and approved the submitted version.

## Funding

The project has received support from Plan on enhancing scientific research in GMU, the Health Economics Association of Guangdong Province (Grant#2023-WJMZ-51) and the Guangdong Basic and Applied Basic Research Foundation (Grant#2021A1515110743).

## Conflict of interest

The authors declare that the research was conducted in the absence of any commercial or financial relationships that could be construed as a potential conflict of interest.

## Publisher’s note

All claims expressed in this article are solely those of the authors and do not necessarily represent those of their affiliated organizations, or those of the publisher, the editors and the reviewers. Any product that may be evaluated in this article, or claim that may be made by its manufacturer, is not guaranteed or endorsed by the publisher.
