# Supplementary material for: Influence of social support on subjective well-being of patients with chronic diseases in China: chain-mediating effect of self-efficacy and perceived stress
Source: Front Public Health. 2023 Jun 21;11:1184711. doi: 10.3389/fpubh.2023.1184711 (PMC10325675; doi:10.3389/fpubh.2023.1184711)
Supplement: Supplementary file 1 [file Data_Sheet_1.pdf]

Supplementary Material

Influence of Social Support on Subjective Well-being of Patients with Chronic Diseases in China: Chain-Mediating Effect of Self-efficacy and Perceived Stress

Zhenni Luo[1,#], Sisi Zhong[1,#], Siyu Zheng[1], Yun Li[1], Yan Guan[1], Weihong Xu[1], Lu Li[1], Siyuan Liu[2], Haozheng Zhou[2],Xuanhao Yin[2], Yibo Wu[3], Diyue Liu[4,*], Jiangyun Chen[5,*]

[1]School of Health Management, Guangzhou Medical University,Xinzao, Panyu District, Guangzhou 511436, China；

[2]School of Public Health, Southern Medical University, Guangzhou510515, China;

[3]School of Public Health, Peking University, Beijing100091, China；

[4]International School of Public Health and One Health，Hainan Medical University, Haikou 571199, China；

[5]Center for WHO Studies and Department of Health Management, School of Health Management of Southern Medical University, No. 1023-1063, Shatai South Road, Baiyun District, Guangzhou510515, China;

*** Correspondence:** D.L.: L15917189871@outlook.com; J.C.: cjy112@i.smu.edu.cn；

1 Supplementary Figures and Tables

1.1 Supplementary Figures

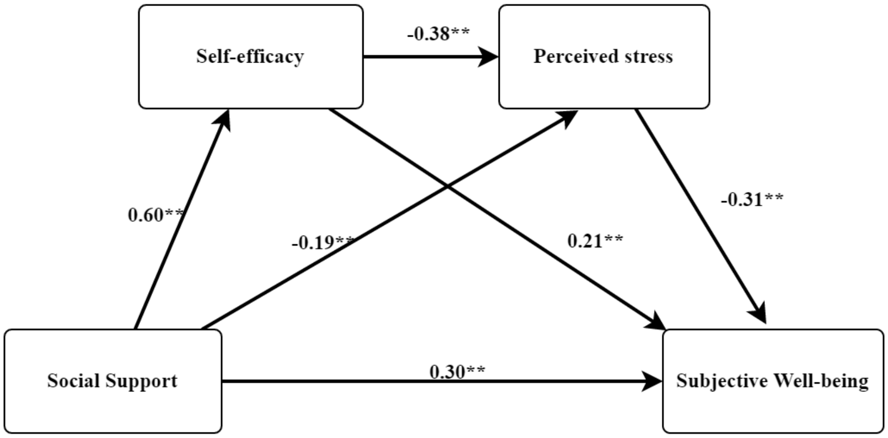

Figure 1. Mediating Model of Self-Efficacy and Perceived Stress Between Social Support and Subjective Well-being

1.2 Supplementary Tables

Table 1. Descriptive Statistics of Research Variables (n = 4657)

**Variable**	***M (SD)***	**MIN–MAX**
Subjective well-being	54.79（21.95）	0-100
Perceived stress	6.39（2.77）	0-16
Social support	14.80（3.41）	3-21
Family support	5.10（1.30）	1-7
Friends support	4.90（1.30）	1-7
Other support	4.79（1.35）	1-7
Self-efficacy	7.58（2.28）	0-12
Level	2.58（0.78）	0-4
Intensity	2.50（0.86）	0-4
Universality	2.51（0.84）	0-4

Table 2. Descriptive Statistics and Correlation Analysis of Variables

Variable	1	2	3	4
1 Subjective well-being	1			
2 Social support	0.563**	1		
3 Self-efficacy	0.546**	0.611**	1	
4 Perceived stress	-0.554**	-0.434**	-0.503**	1

Note：**p* <0.05，***p* <0.01，****p* ＜0.001，similarly hereinafter

Table 3. Moderating Effect of Self-Efficacy and Perceived Stress on Social Support and Well-Being of Patients with Chronic Disease

No.	Variables	*β*(Coeffect)	*SE*	*p*	95% *CI* *LLCI*	*ULCI*	R^2
1	*X→Y*	0.56	0.08	＜0.001	3.43	3.73	0.33
2	*X→M1*	0.60	0.01	＜0.001	0.39	0.42	0.38
3	*X→M2*	-0.19	0.01	＜0.001	-0.18	-0.13	0.31
	M1→M2	-0.38	0.02	＜0.001	-0.50	-0.43	
4	*X→Y*	0.30	0.09	＜0.001	1.75	2.10	0.46
	M1→Y	0.21	0.14	＜0.001	1.71	2.26	
	M2→Y	-0.31	0.10	＜0.001	-2.69	-2.29	

Table 4. Regression Analysis Between Table Variables

Regression equation		Overall fitting index			Significance of regression coefficient	
**Outcome variable**	**Predictor variable**	***R***	***R^2***	***F***	***β***	***t***
Self - efficacy	Gender	0.61	0.38	572.76	0.04	3.37**
	Age				0.00	0.06
	Household registration				-0.03	-2.45*
	Degree of education				0.06	4.02***
	Social support				0.60	51.89***
Perceived stress	Self-efficacy	0.55	0.31	343.24	-0.38	-24.62***
	Social support				-0.19	-12.51***
	Gender				-0.04	-3.21**
	Age				-0.16	-10.464***
	Household registration				0.06	4.06***
	Degree of education				0.01	0.61
Subjective Well-being	Self-efficacy	0.68	0.46	565.42	0.21	14.12***
	Perceived stress				-0.31	-24.25***
	Social support				0.3	21.62***

Sex	-0.01	-1.28
Age	0.05	3.95***
Household registration	-0.00	-0.24
Degree of education	0.01	0.72

Table 5. Mediating Effect Analysis

	Variables	Effect	Boot *SE*	95% CI	
				LLCI	***ULCI***
	Total	1.65	0.07	1.52	1.79
Indirect 1	*X→M1→Y*	0.80	0.07	0.66	0.93
Indirect 2	*X→M1→M2→Y*	0.47	0.03	0.41	0.53
Indirect 3	*X→M2→Y*	0.39	0.04	0.32	0.47
Differences (Δ*B*)	Indirect 1–Indirect 2	0.33	0.08	0.17	0.48
	Indirect 1–Indirect 3	0.41	0.08	0.24	0.57
	Indirect 2–Indirect 3	0.07	0.05	-0.02	0.18

Note**:** *X* = social support, *M1* = self-efficacy, *M2* = perceived stress, and *Y* = subjective well-being.

Attachment 1:

The survey options for chronic diseases in our questionnaire include:

Fracture, cataract, osteoporosis, arthritis, hypertension, stroke (cerebral infarction, cerebral hemorrhage), coronary heart disease, dyslipidemia, diabetes, malignant tumor, benign tumor, chronic respiratory disease (chronic obstructive pulmonary disease/asthma), chronic renal disease, chronic stomach/enteritis, viral hepatitis (e. g., hepatitis b), fatty liver disease, Alzheimer’s disease (senile dementia), Parkinson’s disease, mood disorder (anxiety, depression, etc.), and other chronic diseases.

Attachment 2:

Perceived Social Support Scale (PSSS)

The results of KMO test of PSSS in this study show that KMO value is 0.717. At the same time, the results of Bartlett's spherical test show that the P value of significance is 0.000***, which is significant horizontally, rejecting the original hypothesis, and the variables are correlated, and the factor analysis is effective and the degree is average.

New General Self-Efficacy Scale (NGSES)

The results of KMO test of NGSES in this study show that KMO value is 0.754. At the same time, the results of Bartlett's spherical test show that the P value of significance is 0.000***, which is significant horizontally, rejecting the original hypothesis, and the variables are correlated, and the factor analysis is effective and the degree is average.

Well-being Index Scale (WHO-5)

The results of KMO test of WHO-5 in this study show that KMO value is 0.893. At the same time, the results of Bartlett's spherical test show that the P value of significance is 0.000***, which is significant at the horizontal level, rejecting the original hypothesis, and the variables are correlated, and the factor analysis is effective and the degree is appropriate.

Perceived Stress Scale (PSS-4)

In this study, the KMO test of the PSS-4 showed that the KMO value was 0.61. At the same time, the results of Bartlett's spherical test show that the P value of significance is 0.000***, which is significant at the horizontal level, rejecting the original hypothesis, and the variables are correlated, and the factor analysis is effective and the degree is acceptable.
